# Genomic Insights into Methanotrophy: The Complete Genome Sequence of Methylococcus capsulatus (Bath)

**DOI:** 10.1371/journal.pbio.0020303

**Published:** 2004-09-21

**Authors:** Naomi Ward, Øivind Larsen, James Sakwa, Live Bruseth, Hoda Khouri, A. Scott Durkin, George Dimitrov, Lingxia Jiang, David Scanlan, Katherine H Kang, Matt Lewis, Karen E Nelson, Barbara Methé, Martin Wu, John F Heidelberg, Ian T Paulsen, Derrick Fouts, Jacques Ravel, Hervé Tettelin, Qinghu Ren, Tim Read, Robert T DeBoy, Rekha Seshadri, Steven L Salzberg, Harald B Jensen, Nils Kåre Birkeland, William C Nelson, Robert J Dodson, Svenn H Grindhaug, Ingeborg Holt, Ingvar Eidhammer, Inge Jonasen, Susan Vanaken, Terry Utterback, Tamara V Feldblyum, Claire M Fraser, Johan R Lillehaug, Jonathan A Eisen

**Affiliations:** **1**The Institute for Genomic Research, RockvilleMarylandUnited States of America; **2**Center of Marine Biotechnology, BaltimoreMarylandUnited States of America; **3**Department of Biology, University of BergenBergenNorway; **4**Department of Molecular Biology, University of BergenBergenNorway; **5**Department of Biology, Johns Hopkins UniversityBaltimore, MarylandUnited States of America; **6**National Institutes of Health, National Human Genome Research InstituteBethesda, MarylandUnited States of America; **7**Department of Informatics, University of BergenBergenNorway; **8**George Washington University Medical Center, WashingtonDistrict of ColumbiaUnited States of America; **9**Department of Computer Science, Johns Hopkins UniversityBaltimore, Maryland United States of America

## Abstract

Methanotrophs are ubiquitous bacteria that can use the greenhouse gas methane as a sole carbon and energy source for growth, thus playing major roles in global carbon cycles, and in particular, substantially reducing emissions of biologically generated methane to the atmosphere. Despite their importance, and in contrast to organisms that play roles in other major parts of the carbon cycle such as photosynthesis, no genome-level studies have been published on the biology of methanotrophs. We report the first complete genome sequence to our knowledge from an obligate methanotroph, Methylococcus capsulatus (Bath), obtained by the shotgun sequencing approach. Analysis revealed a 3.3-Mb genome highly specialized for a methanotrophic lifestyle, including redundant pathways predicted to be involved in methanotrophy and duplicated genes for essential enzymes such as the methane monooxygenases. We used phylogenomic analysis, gene order information, and comparative analysis with the partially sequenced methylotroph Methylobacterium extorquens to detect genes of unknown function likely to be involved in methanotrophy and methylotrophy. Genome analysis suggests the ability of M. capsulatus to scavenge copper (including a previously unreported nonribosomal peptide synthetase) and to use copper in regulation of methanotrophy, but the exact regulatory mechanisms remain unclear. One of the most surprising outcomes of the project is evidence suggesting the existence of previously unsuspected metabolic flexibility in *M. capsulatus,* including an ability to grow on sugars, oxidize chemolithotrophic hydrogen and sulfur, and live under reduced oxygen tension, all of which have implications for methanotroph ecology. The availability of the complete genome of M. capsulatus (Bath) deepens our understanding of methanotroph biology and its relationship to global carbon cycles. We have gained evidence for greater metabolic flexibility than was previously known, and for genetic components that may have biotechnological potential.

## Abstract 2

Methanotrophs are bacteria that use methane as a sole carbon source. The genome sequence of *Methylococcus capsulatus* deepens our understanding of methanotroph biology and its relationship to global carbon cycles

## Introduction

Methanotrophic bacteria such as Methylococcus capsulatus are responsible for the oxidation of biologically generated methane (Soehngen 1906), and they are therefore of great environmental importance in reducing the amount of this greenhouse gas released to the Earth's atmosphere. Atmospheric methane levels have been increasing over the last 300 years, and it is thought that this is mostly due to human activity. Methane is a very effective greenhouse gas; it has been estimated that methane contribution to climate change is about 26 times that of carbon dioxide (mole for mole) (Ehalt 1974; Ehalt and Schmidt 1978; Lelieveld et al. 1993). The effect is further amplified by the reduction of hydroxyl radical concentrations due to increasing atmospheric methane levels; these radicals oxidize methane photochemically, thus their loss from the atmosphere increases the persistence of methane (Lelieveld et al. 1993).

**Figure 1:**
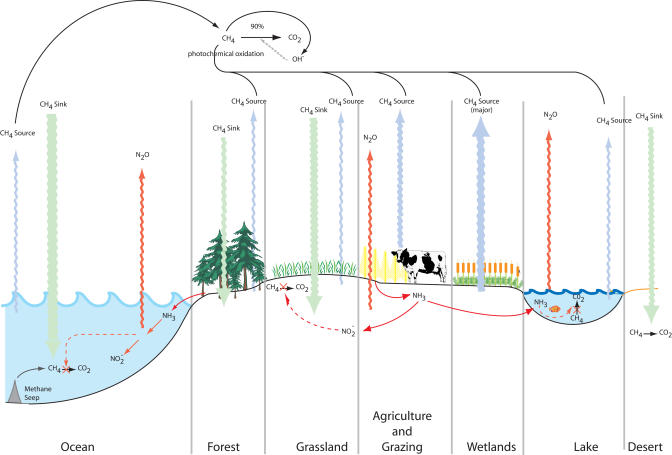
Global Methane Cycle Methane is oxidized either photochemically in the atmosphere or biologically in terrestrial and aquatic systems. The ocean, grasslands, and desert form major methane sinks, whereas wetlands, agricultural and grazing lands, and other anthropogenic sources such as landfills, are major sources. The cow depicted in the figure represents diverse ruminants. Anthropogenic inputs of nitrogen in the form of ammonia compete for MMOs, reducing methane oxidation and leading to the formation of nitrous oxide, another greenhouse gas.

Biological methane oxidation is known to occur aerobically in both terrestrial and aquatic habitats, and anaerobically in sediments and anoxic salt water. It acts on methane biologically generated in situ and on methane scavenged from the atmosphere (Figure 1). Deep-sea environments such as cold gas seeps and hydrothermal vents exhibit a photosynthesis-independent food chain based on methanotrophs and chemolithotrophs, some of which form symbiotic partnerships with invertebrates (e.g., Cavanaugh et al. 1987). Methanotrophs are also able to metabolize or co-metabolize xenobiotic compounds, including chlorinated solvents such as trichloroethylene, and hence have potential as bioremediation tools (Large and Bamforth 1988).

Distribution of methanotrophy within the Bacteria is currently thought to be relatively limited, being found so far in only 11 genera of the Proteobacteria. These methanotrophs are classified into two types, based primarily on their phylogenetic relationships but also on differences in their physiology and internal membrane structure. Type I methanotrophs (including *Methylococcus*), which are all members of the Gammaproteobacteria, utilize ribulose monophosphate (RuMP) as the primary pathway for formaldehyde assimilation, whereas those of type II, which are all Alphaproteobacteria, use the serine pathway (Hanson and Hanson 1996).

M. capsulatus is classified as an obligate methanotroph (Whittenbury et al. 1970); methane is oxidized via methanol to formaldehyde, which is then assimilated into cellular biomass or further oxidized to formate and CO_2_ for energy production. The conversion of methane to biomass by M. capsulatus has been exploited for large-scale commercial production of microbial proteins by fermentation (Skrede et al. 1998).

The powerful tool of whole-genome sequencing has been applied to microorganisms that carry out other important components of the carbon cycle, such as photosynthesis (e.g., Eisen et al. 2002; Dufresne et al. 2003) and methanogenesis (Bult et al. 1996; Smith et al. 1997; Slesarev et al. 2002), but there is a paucity of genomic information on the methanotrophs, which are equally important contributors to global carbon cycles. Many insights into methylotrophy have been gained from the recently available partial genome sequence of Methylobacterium extorquens (AM1) (Chistoserdova et al. 2003, 2004), but this organism, like other nonmethanotrophic methylotrophs, is limited to the oxidation of C1 compounds other than methane. We undertook the whole-genome sequencing of M. capsulatus (Bath) to obtain a better understanding of the genomic basis of methanotrophy, a globally important microbial process.

## Results/Discussion

### Genome Properties

The genome of M. capsulatus (Bath) comprises a single circular molecule of 3,304,697 bp. General features of the genome and its 3,120 predicted coding sequences (CDSs) are summarized in Table 1. GC skew (Lobry 1996) and oligoskew (Salzberg et al. 1998b) analyses were used to identify a putative origin of replication, and basepair 1 was assigned upstream of the glucose-inhibited division protein A*(gidA)* gene(MCA0001), adjacent to the chromosome-partitioning proteins encoded by *gidB, parA,* and *parB* and the operon that encodes F_1_F_0_ ATP synthase.

**Table 1:**
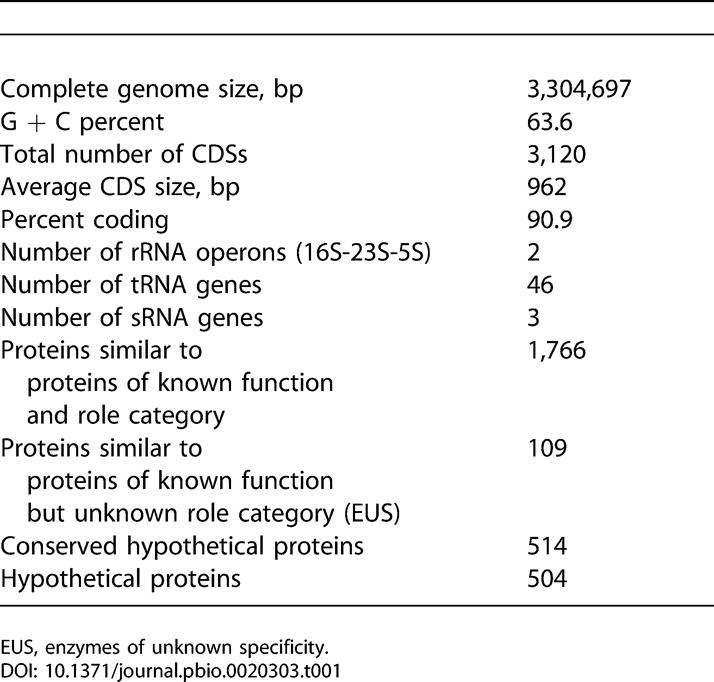
General Features of the M. capsulatus (Bath) Genome

EUS, enzymes of unknown specificity

The M. capsulatus (Bath) genome contains 51 identifiable insertion sequence elements from various families (Chandler and Mahillon 2002). As is found in other sequenced bacterial genomes, many of these elements share higher intra- than intergenome similarity. This suggests several possible mechanisms: expansion of these elements since their introduction into the M. capsulatus (Bath) genome, repeated cycles of duplication and subsequent deletion, or gene conversion. Twenty elements belonging to the IS4 family of insertion sequences (Chandler and Mahillon 2002) encode a 366-amino-acid transposase with 100% amino acid sequence conservation between copies. One copy (MCA1197) is found within the soluble methane monooxygenase (sMMO) operon, although not in all sequenced clones, suggesting that this element is highly mobile. Other examples of insertion of this element include disrupted genes encoding tRNA pseudouridine synthase (split into two putative CDS—MCA1311 and MCA1313) and an exopolysaccharide export protein (split into MCA1176 and MCA1178).

Two putative prophages (one of approximately 58.5 Kbp, spanning from MCA2632 to MCA2689 and the other, a Mu-phage-like element of approximately 45 Kbp spanning from MCA2900 to MCA2959) were identified in the genome. The Mu-like prophage is unusual in encoding an intein within F (Mu gp30), a cofactor in head assembly. This intein region is most similar to inteins present in several archaeal translation initiation factor IF-2 sequences from the genera *Pyroccocus* and *Methanococcus,* sharing 41% sequence identity and 62% sequence similarity with the Pyrococcus horikoshii intein. The intein lacks a recognizable endonuclease sequence and appears degenerate compared to the archaeal IF-2 intein, casting doubt on its ability to be mobile. If functional, the presence of the intein in this protein suggests that head morphogenesis could be regulated by conditions that influence the rate of intein excision. Another intein sharing sequence similarity with archaeal inteins was identified in the gene encoding ribonucleotide reductase (MCA2543). Inteins are rare, but when present are often found in genes associated with nucleotide metabolism, such as ribonucleotide reductases.

Bacteriophages have been important tools for genetic manipulation of bacterial genomes, and such tools are currently lacking for M. capsulatus (Bath). The M. capsulatus (Bath) Mu-like prophage could be engineered to resemble the Mu derivatives, which have been excellent tools for random mutagenesis in other species (Casadaban and Cohen 1979). Conditional protein splicing via inteins is used as a tool for protein engineering, drug therapy, and vaccine development (Humphries et al. 2002; Mootz et al. 2003; Nyanguile et al. 2003). The putative inteins could be designed as a tool either for generating protein material for vaccination of salmon (or other animals feeding on M. capsulatus proteins) or for manipulating M. capsulatus live vaccine vectors.

### Metabolism and Transport: Genomic Basis of the Methanotrophic Lifestyle

**Figure 2:**
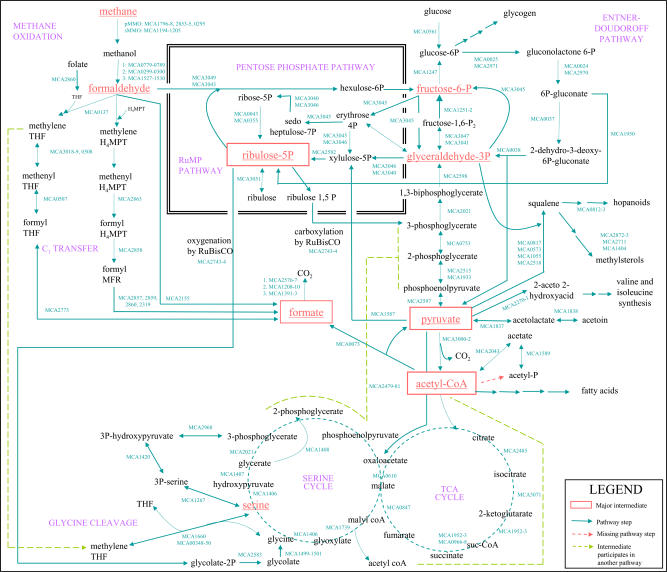
Predicted Central Metabolic Pathways of M. capsulatus Genomic information was used to predict the flow of carbon from methanotrophy pathways into carbon fixation pathways, and thence into glycolysis/gluconeogenesis and the TCA cycle. Locus names are indicated next to key steps. Some intermediates are omitted.

We have attempted to predict central metabolic pathways in M. capsulatus (Bath), including the methane oxidation pathway, mechanisms for carbon fixation, glycolytic and gluconeogenic conversions, and the tricarboxylic acid (TCA) cycle, from analysis of the genome data. These pathways, together with those known from previous studies, are depicted in Figure 2, along with the locus numbers for predicted enzymes. Some of these pathways have not been experimentally verified, so we present Figure 2 as a hypothesis of metabolic activity in M. capsulatus (Bath) that is based on available genome data.

#### Methane oxidation

Methanotrophs are unique in their possession of methane monooxygenases (MMOs), which catalyze the first step of methane oxidation (Figure 2). M. capsulatus is known to possess both a particulate membrane-bound form, pMMO (detected by centrifugation studies and encoded by *pmo*), and a soluble form, sMMO (encoded by *mmo*), and these enzymes have been extensively studied (Murrell 1994; Nguyen et al. 1998; Stolyar et al. 1999; Coufal et al. 2000; Murrell et al. 2000a, 2000b; Whittington and Lippard 2001). The pMMO was previously known to consist of three subunits encoded by *pmoCAB* (Zahn and DiSpirito 1996); two complete copies of *pmoCAB* and a third copy of *pmoC (pmoC3)* were previously identified (Stolyar et al. 1999). Our genomic analysis suggests the pMMO genes have been recently duplicated (Table 2). The *pmoC3* gene is located in a putative operon with three additional genes of unknown function, and we can speculate that these three are also related to methane oxidation.

**Table 2:**
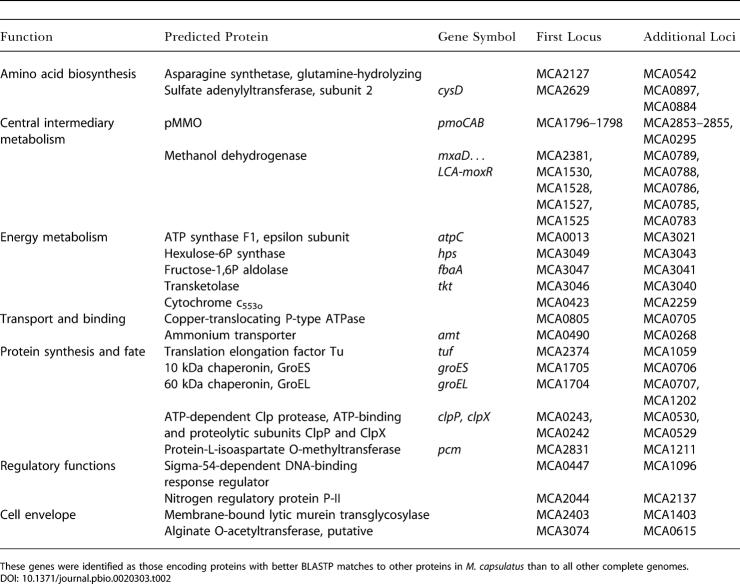
Selected Putative Lineage-Specific Gene Duplications in M. capsulatus (Bath)

These genes were identified as those encoding proteins with better BLASTP matches to other proteins in M. capsulatus than to all other complete genomes

Only one chromosomal locus for the components of sMMO was identified (*mmoXYB–*transposase*–mmoZDC–*hypothetical protein*–mmoGQSR* [MCA1194–1205]). The transposase (MCA1197) is oriented in the same direction as the *mmo* genes and, thus, may be transcribed under sMMO-promoting growth conditions. The functions of these sMMO components have been previously determined (Stainthorpe et al. 1990; Nielsen et al. 1997; Coufal et al. 2000; Merkx and Lippard 2002; Csaki et al. 2003).

#### Methanol oxidation

Methanol is available to M. capsulatus from the oxidation of methane and presumably also from exogenous sources (e.g., pectin and lignin degradation) (Hanson and Hanson 1996), and its oxidation is catalyzed by methanol dehydrogenases (Anthony and Williams 2003). We have detected three sets of genes encoding homologs of the structural components of methanol dehydrogenase (homologs of MxaF and MxaI) and the proteins required for its catalytic function *(*homologs of MxaJGRACKLD*)* (Figure 2). There is one large cluster of genes (MCA0779–0790) including homologs of *mxaFJGIRACKLD,* which probably encodes a heterodimeric methanol dehydrogenase, as is found in other methylotrophs (Amaratunga et al. 1997a, 1997b). Also like other methylotrophs, M. capsulatus (Bath) contains a second methanol dehydrogenase–like cluster, *mxaFJ,* (MCA0299–0300) lacking a homolog of *mxaI* that normally encodes the small subunit of methanol dehydrogenase. The function of *mxaJ* is unknown, and it is not clear whether this *mxaFJ* cluster encodes components active in methanol oxidation. There is a third cluster of genes required for methanol dehydrogenase synthesis, *mxaACKL* (MCA1527–1530).

#### Formaldehyde and formate oxidation

Formaldehyde is the substrate for carbon fixation through the RuMP pathway in M. capsulatus (Attwood and Quayle 1984) and thus is an important intermediate in both catabolism and anabolism (Figure 2). However, formaldehyde is also highly toxic, and the cell needs to tightly control its production (Attwood and Quayle 1984). We were able to compare the results of genomic analysis with previously reported pathways for formaldehyde oxidation in M. capsulatus. A membrane-bound pyrroloquinoline quinine protein is known to be the major formaldehyde dehydrogenase under high-copper growth conditions, while a soluble NAD(P)^+^-linked formaldehyde dehydrogenase is active when copper concentrations are low (Zahn et al. 2001). Other previously characterized formaldehyde oxidation pathways in M. capsulatus include the cyclic pathway that uses enzymes of the RuMP pathway (Strom et al. 1974), and the tetrahydromethanopterin (THMPT)-linked pathway (Vorholt 2002).

Genome analysis showed the previously determined N-terminal sequence of the pyrroloquinoline quinine–containing formaldehyde dehydrogenase (Zahn et al. 2001) to match MCA2155, a protein resembling a sulfide-quinone reductase (SQR), on the basis of both its motifs and its phylogenetic relationship to other SQRs. Zahn et al. (2001) reported homology of their N-terminal sequenced enzyme with SQRs from other bacteria, but were unable to obtain experimental evidence for quinone reductase activity. In the absence of this evidence, we have annotated the gene as a formaldehyde dehydrogenase.

We found that the previously sequenced 63-kDa subunit of NAD(P)^+^-linked formaldehyde dehydrogenase (Tate and Dalton 1999) best matches the N-terminal of the large subunit of methanol dehydrogenase (MCA0779), although the sequences differ slightly. The sequence of modifin, the 8.6-kDa subunit thought to confer substrate specificity to the enzyme (Stirling and Dalton 1978; Tate and Dalton 1999), could not be clearly identified in the genome. A recent paper (Adeosun et al. 2004) helps resolve this conflict between the genome and these previous results; apparently the NAD(P)^+^-linked activity is due to an artefactual mixture of methanol dehydrogenase and methylene dehydrogenase.

We also identified components of the RuMP (Entner-Douderoff pathway)-linked and THMPT-linked pathways of formaldehyde oxidation (Figure 2). In addition to the formaldehyde oxidation systems described above, genome analysis suggests an additional complete tetrahydrofolate (THF)-linked pathway (Figure 2) previously undescribed in *M. capsulatus,* as was recently found alongside the THMPT pathway in M. extorquens (Vorholt 2002; Chistoserdova et al. 2003). In M. extorquens, the THF pathway is thought to play a role in assimilation of carbon from both formaldehyde and formate, while THMPT is involved in catabolic oxidation of formaldehyde to formate using the same enzymes used for methanogenesis; free formaldehyde is thought to be the substrate for hexulose-6-phosphate synthase in the RuMP pathway (Strom et al. 1974; Vorholt 2002). M. capsulatus possesses homologs to genes encoding proteins in all of these pathways; therefore, it may have the capability to assimilate/detoxify formaldehyde in the same way.

We have identified three previously undescribed homologs of formate dehydrogenases (MCA2576–2577, MCA1208–1210, and MCA1391–1393). Multiple formate dehydrogenases occur in other bacteria (Sawers 1994; Chistoserdova et al. 2004); in *M. extorquens,* all three are expendable, indicating that this last step in methane oxidation plays a minor role and that formate can be dissimilated in other ways (Chistoserdova et al. 2004). The importance of the formate dehydrogenases in M. capsulatus remains to be determined.

The genomic redundancy in the set of methane oxidation pathways suggests that M. capsulatus exploits different systems under variable environmental conditions (e.g., copper levels). It is plausible that M. capsulatus balances its requirement for formaldehyde-derived carbon and reducing power with the toxicity of formaldehyde by taking advantage of three enzymes for formate oxidation and multiple pathways for formaldehyde oxidation under different environmental conditions. This redundancy has implications for future attempts to manipulate the genes of this pathway; simple knockouts may not be achievable.

#### Carbon fixation

M. capsulatus is known to assimilate formaldehyde through the RuMP pathway (Strom et al. 1974) (Figure 2). Genomic analysis suggests that RuMP components have experienced a lineage-specific duplication (Table 2); there is a 5,267-bp identical direct repeat centered around the transaldolase gene that contains the genes for hexulose-6-phosphate isomerase*,* hexulose-6-phosphate synthase*,* fructose-1,6-phosphate aldolase*,* and transketolase*.* There is evidence that the RuMP pathway is also used for gluconeogenesis (see below); this dual function may have been facilitated by the redundancy resulting from this tandem duplication. The M. capsulatus (Bath) genome appears to contain some parts of the alternative serine pathway of formaldehyde assimilation (Figure 2), including a candidate for the key serine cycle enzyme malyl-CoA lyase (MCA1739). Activities associated with this pathway have been reported as “sometimes” present (Hanson and Hanson 1996). However, the majority of enzymes with a putative role in the serine cycle also have putative roles in other metabolic pathways (e.g., there are candidate genes encoding proteins that may be able convert malate to malyl-CoA—MCA1740–1741 are similar to the two subunits of malate Co-A ligase from M. extorquens [Chistoserdova and Lidstrom 1994] but are also similar to the two subunits of succinyl Co-A synthases). In addition, the genome apparently lacks any good candidates for other steps in the serine cycle such as the conversion of phosphoenolpyruvate to oxaloacetate (i.e., phosphoenolpyruvate carboxylase). The latter enzyme may be circumvented by a likely oxaloacetate decarboxylase (MCA2479–2481), which converts pyruvate to oxaloacetate (Figure 2). It is possible that M. capsulatus fixes formaldehyde through the serine cycle as far as oxaloacetate (Figure 2).

It appears that the Calvin cycle operates with transketolase (MCA3040 and MCA3046), reversibly converting glyceraldehyde-3-phosphate to xylulose-5-phosphate, bypassing the typical ribose-5-phosphate to fructose-6-phosphate segment (Figure 2); cell suspensions of M. capsulatus grown on methane do not exhibit seduheptulose-1,7-bisphosphatase activity (Strom et al. 1974), and a gene encoding this enzyme was not identified in the genome sequence.

#### Redundancy in serine and glycogen biosynthesis pathways

Serine is an important intermediate in M. capsulatus metabolism, and genomic evidence suggests three potential pathways for serine synthesis not previously described in *M. capsulatus:* a phosphorylated pathway from glycerate-3-phosphate, a nonphosphorylated pathway from glycerate, and a nonphosphorylated pathway from glycolate-2-phosphate (Ho and Saito 2001) (Figure 2).

Homologs of genes encoding enzymes predicted to catalyze the three steps in the phosphorylated pathway (3-phosphoglycerate dehydrogenase, phosphoserine aminotransferase, and phosphoserine phosphatase) are present, but the latter two may have alternate functions in vitamin B6 biosynthesis (Lam and Winkler 1990) or as homoserine kinases. Genes normally encoding homoserine kinases *(thrB* and *thrH)* were not identified in the M. capsulatus (Bath) genome, and phosphoserine phosphatase may perform this function as described in Pseudomonas aeruginosa (Patte et al. 1999).

The nonphosphorylated pathway is not well characterized at the molecular level, but it is known to be initialized by the dephosphorylation of phosphoglycerate to glycerate (Ho and Saito 2001); subsequently, glycerate is oxidized to hydroxypyruvate and hydroxypyruvate is transaminated to serine (Figure 2). The genome encodes a homolog of glycerate kinase, a 2-hydroxyacid dehydrogenase that may function as a hydroxypyruvate reductase, and a serine-glyoxylate aminotransferase, which may also have serine-pyruvate transaminase activity. Genes encoding these three enzymes appear to be organized in an operon (MCA1406–1408), supporting their proposed roles in serine formation from phosphoglycerate. The M. capsulatus (Bath) genome also encodes a putative phosphoglycerate mutase (MCA0753), to interconvert 3- and 2-phosphoglycerate (Figure 2), allowing the organism to carry out either the phosphorylated or nonphosphorylated pathway.

In the third pathway, M. capsulatus may derive glycolate-2-phosphate from the oxygenation reaction of ribulose bisphosphate carboxylase (MCA2743–2744, previously identified by Baxter et al. [2002]), convert it to glycine, which is split into carbon dioxide, ammonia, and methylene-tetrahydrofolate (Figure 2). A second glycine molecule and methylene-tetrahydrofolate ligate to form serine. There is experimental evidence (Taylor et al. 1981) for this pathway of glycolate-2-phosphate assimilation, which resembles that of plants.

#### Evidence for novel gluconeogenesis pathways and a complete TCA cycle

A key enzyme in gluconeogenesis is fructose-1,6-bisphosphatase, which catalyzes the irreversible dephosphorylation of fructose-1,6-phosphate to fructose-6-phosphate; genes encoding this enzyme are absent. However, there are three potential alternative pathways for gluconeogenesis, previously unknown in this organism (Figure 2). First, there is a transaldolase homolog (MCA3045) that may convert glyceraldehyde-3-phosphate directly to fructose-6-phosphate. Second, there is a putative phosphoketolase (MCA1587), which can condense pyruvate and glyceraldehyde-3-phosphate into xylulose-5-phosphate, which in turn is fed into the ribulose-5-phosphate pool for eventual formation of glucose-6-phosphate through the pentose phosphate pathway. Third, hydrolysis of fructose-1,6-bisphosphate to fructose-6-phosphate by a pyrophosphate-dependent 6-phosphofructokinase and a pyrophosphatase may occur, as was recently proposed in *Nitrosomonas* (Chain et al. 2003).

An incomplete TCA cycle lacking 2-oxoglutarate dehydrogenase activity has been found in nearly all type I methanotrophs, including M. capsulatus (Hanson and Hanson 1996). However, genes encoding homologs of 2-oxoglutarate dehydrogenase are present (MCA1952 and MCA1953). Thus, a complete TCA-cycle might operate in M. capsulatus, not under methane oxidation, but under other conditions. Lack of experimental evidence precludes speculation as to the nature of these conditions; however, catabolite repression (Wood et al. 2004) may play a role here. Another type I methylotroph, *Methylomonas* sp. (761), uses a complete TCA cycle to grow on glucose as its sole carbon and energy source (Zhao and Hanson 1984); M. capsulatus may utilize the same mechanism as carbon is stored as glycogen. Consistent with its autotrophic lifestyle, M. capsulatus possesses only a limited array of membrane transporters for organic carbon compounds. However, although M. capsulatus is not known to utilize any sugars (although in the Texas strain they have been reported to support growth), one complete (MCA1941–1944) and one partial (MCA1924) ATP-binding casette (ABC) family transporter with predicted specificity for sugar uptake were identified. Additionally, components of transporters for peptides (MCA1264 and MCA1268), carboxylates (MCA1872), and a variety of amino acids (e.g., MCA0840) are present.

#### Diversity of nitrogen metabolism

M. capsulatus (Bath) is able to fix atmospheric nitrogen (Murrell and Dalton 1983), conferring an advantage in environments where fixed nitrogen is limiting, and the structural genes for nitrogenase *(nifH, nifD,* and *nifK)* were previously shown to be contiguous (Oakley and Murrell 1991), as they are in other nitrogen fixers. Genome analysis extends this contiguous region to include the genes *nifE, nifN,* and *nifX,* which are involved in synthesis of the nitrogenase iron-molybdenum cofactor (MCA0229–0239); this organization has been found in Chlorobium tepidum and some nitrogen-fixing methanogenic Archaea. Two 2Fe-2S ferredoxins (MCA0232 and MCA0238) and two genes identified as conserved hypotheticals (MCA0236–0237) are interspersed with the *nif* genes in the same orientation. The conserved hypothetical genes share the highest sequence similarity with genes from other organisms capable of nitrogen fixation, suggesting that they also have a role in this process.

M. capsulatus exhibits considerable versatility in its combined nitrogen conversions, including nitrification and denitrification. Ammonia is oxidized to nitrite by both pMMO and sMMO because of their lack of substrate specificity (Colby et al. 1977; Dalton 1977); the absence of a separate ammonia monooxygenase, and the redundancy of MMOs, suggests that the MMOs are the sole nitrification enzymes active in M. capsulatus. Four predicted ammonium transporters were identified (MCA0268, MCA0490, MCA1581, and MCA2136), suggesting that ammonium is an important nitrogen source for M. capsulatus. Methane oxidation is inhibited by the presence of ammonia and ammonia oxidation is inhibited by methane (Whittenbury et al. 1970), and input of ammonia to wetland systems (e.g., through fertilizer runoff) may have significant effects on the consumption of biogenic methane by methanotrophs in these systems. It is also interesting to note that, in general, ammonia oxidation produces small amounts of nitrous oxide, which is also a greenhouse gas.

#### Electron transport complement suggests unexpected metabolic flexibility

The M. capsulatus (Bath) genome has a relatively large complement of putative c-type cytochromes; 57 proteins containing a heme-binding motif were identified, and 23 of these contain two or more heme-binding motifs. Analysis of the genome reveals electron transport components previously known to be associated with the methane oxidation pathway, such as cytochrome C_L_ (MCA0781), a specific electron acceptor for methanol dehydrogenase. Other novel electron transport components are encoded in several physical locations on the chromosome; there is genomic evidence for chemolithotrophy and the ability to live at a variety of oxygen tensions.

The genome encodes three predicted hydrogenases: (a) a multisubunit formate hydrogenlyase (MCA1137–1142), most likely involved in the conversion of formate to dihydrogen and carbon dioxide; (b) a soluble cytoplasmic NAD-reducing hydrogenase (MCA2724–2726), which transfers electrons to NAD^+^; and (c) a membrane-bound Ni-Fe hydrogenase (MCA0163–0165). Activity of two hydrogenases (one soluble and one membrane-bound), and the role of molecular hydrogen in driving MMOs, was previously reported (Hanczar et al. 2002). The membrane-bound Ni-Fe hydrogenase was previously sequenced (Csaki et al. 2001). The presence of these two hydrogenases suggests that M. capsulatus is able to capture and oxidize hydrogen that is generated either exogenously or as a by-product of the ATP-dependent reaction of nitrogenase, and recycle it into the electron transport chain. Microorganisms that undergo fermentative metabolism are likely to be encountered in the habitat of M. capsulatus (e.g., soils) and could supply exogenous hydrogen for chemolithotrophic oxidation. The removal of this hydrogen by M. capsulatus metabolism may aid in driving these reactions forward and hence constitute a syntrophic partnership.

Nitrogen fixation requires reducing power, which in aerobes can be supplied by reduced flavodoxin or ferredoxins. There is one candidate flavodoxin present (MCA1697) in the M. capsulatus (Bath) genome; however, two ferredoxins (MCA0238 and MCA0232) physically located within a cluster of genes encoding proteins involved in nitrogen fixation (see Nitrogen Metabolism section above) more likely serve in this capacity. In aerobes, these carriers are usually reduced by NADH/NADPH, although reverse electron transport could be involved in this organism.

The M. capsulatus (Bath) genome encodes homologs of a two-subunit high oxygen-affinity cytochrome d (MCA1105 and MCA1106), which suggests the ability to live under microaerophilic conditions. This evidence for life at low oxygen tensions is supported by the presence of enzymes indicative of fermentative activity (Table 3). Further support for anaerobiosis is provided by a putative large c-type cytochrome (MCA2189) that contains 17 heme groups and is located adjacent to several hypothetical proteins, including an oxidoreductase and an alkaline phosphatase important to the central metabolism of phosphorous compounds. This cytochrome has significant matches only to high molecular-weight cytochromes in the metal-ion reducers *Shewanella oneidensis, Desulfovibrio vulgaris,* and *Geobacter sulfurreducens,* suggesting that M. capsulatus may have the ability to undergo metabolism at a lower redox potential than previously known. This large protein is most likely localized in the periplasm, as indicated by its signal peptide. The ability to oxidize methane under reduced oxygen tensions would provide an advantage to M. capsulatus in allowing it to be physically closer to environments in which methane is biologically generated.

**Table 3:**
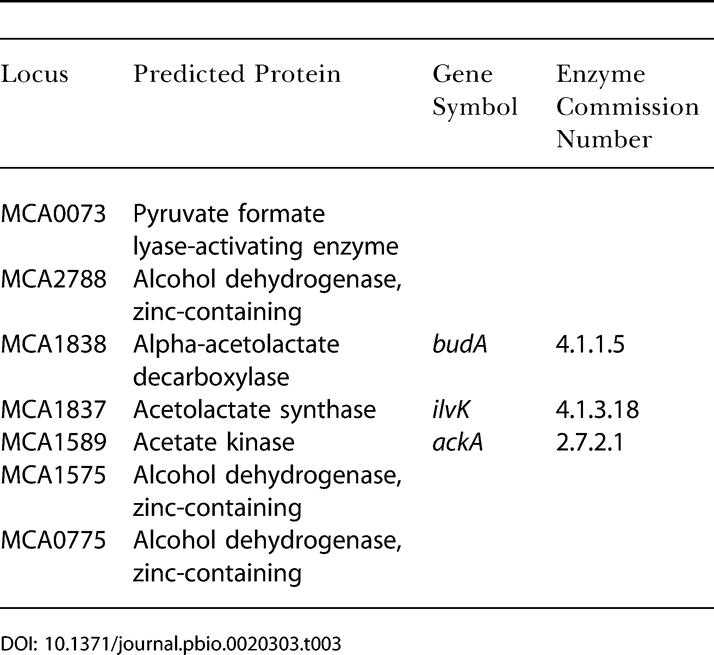
Putative Enzymes Associated with Fermentation

The M. capsulatus (Bath) genome includes a region of approximately 25 kb (MCA0421–0443) encoding novel proteins related to energy metabolism (c-type cytochromes, a c-type cytochrome biogenesis protein, a novel gene possessing a flavodoxin domain, two proteins that may be involved in heme transport, and an undescribed Fe-S binding protein) and hypothetical proteins. The same 25-kb region contains six multiheme c-type cytochromes that are members of the cytochrome c_553o_ family. This previously described family is unique to M. capsulatus (Bath); genome analysis has widened our knowledge of this family from three (Bergmann et al. 1999) to six members (MCA0338, MCA0421, MCA0423, MCA0424, MCA2259, and MCA2160). The redundancy in cytochrome c_553o_ proteins suggests a more complex and plastic electron transport capability than previously known.

A novel monoheme c-type cytochrome (MCA1187) has eight transmembrane-spanning regions and is located near another c-type cytochrome and a proton-translocating pyrophosphatase (a transmembrane-spanning protein pump important in establishing electrochemical gradients). This configuration suggests that the monoheme protein has a role in ATP production via chemiosmosis. Two other monoheme CDSs (MCA2188 and MCA2196) are members of a paralogous family similar to a cytochrome found in *G. sulfurreducens;* one has a signal for twin arginine transport (for export from the cytoplasm) and the second a signature for fumarate lyase. The genome also encodes homologs to the putidaredoxin family of ferredoxins.

#### Genomic evidence for anaerobic synthesis of unique fatty acids

The membrane phospholipids of methanotrophs are unique, with mono-unsaturated fatty acids consisting of a series of even- and odd-numbered positional isomers of both the *cis* and *trans* configuration (Makula 1978). Our analysis supports the existence of an anaerobic mechanism for unsaturated fatty acid synthesis in *M. capsulatus,* as proposed by Jahnke and Diggs (1989) on the basis of biochemical data*.* A predicted enzyme 3-hydroxydecanoyl-ACP-dehydratase (MCA2878) appears to catalyze an alternative dehydratase reaction at the C_10_ level of fatty acid synthesis, followed by a synthase reaction carried out by 3-oxoacyl-acyl carrier protein (ACP) synthase (MCA2879), resulting in *cis*-vaccenate (18:1, *cis*-Δ11). *Trans*-unsaturated acids are obtained by isomerization of preformed *cis*-unsaturated fatty acids, to control membrane fluidity; putative fatty acid *cis*/*trans*-isomerases were identified in the genome (MCA1585 and MCA1806)*.*

#### Sterol and hopanoid biosynthesis: evidence for the mevalonate-independent pathway

M. capsulatus is one of only a few prokaryotes known to synthesize sterols de novo (Bird et al. 1971), and it is thought that they have a role in maintaining membrane fluidity in response to changes in environmental temperature (Jahnke 1992). The main cholesterols in M. capsulatus (Bath) are methylated; genome analysis indicated four putative proteins involved in the conversion of squalene to 4,4-dimethylcholest-8(14)-en-3β-ol (MCA2872, MCA2873, MCA2711, and MCA1404).

Squalene is also a precursor for hopanoid synthesis (Figure 2); homologs of squalene hopene cyclase, squalene synthase, and other enzymes leading to hopanoid synthesis were identified (MCA0812, MCA0813, and MCA2873). Acetyl-CoA is usually the starting point for synthesis of hopanoids and sterols. However, with the exception of the final step catalyzed by geranyl-*trans*-transferase, the mechanism for converting acetyl-CoA to squalene (the first major intermediate) is not apparent from genome analysis. Instead, M. capsulatus (Bath) contains genes for the alternative mevalonate-independent pathway from glyceraldehyde-3-phosphate and pyruvate (MCA0817, MCA0573, MCA1055, and MCA2518), so it is possible that the organism employs the same squalene synthesis pathway found in plants and many Gram-negative bacteria (Rohdich et al. 2003).

### Environmental Sensing, Response, and Survival

#### Copper homeostasis, scavenging, and transport.

Copper is known to be important in the regulation of MMO activity; high copper concentrations are essential for the formation of extensive intracytoplasmic membranes and pMMO activity, and copper is thought to play an active role in both the catalytic site and the electron transport chain (Nguyen et al. 1994; Semrau et al. 1995; Basu et al. 2003). In contrast, sMMO activity is inhibited by copper; synthesis of sMMO may allow methanotrophs to survive in copper-limited environments where pMMO cannot be active. Two distinct copper transporting systems have been identified in other bacteria, a P-type ATPase (the Cop system) and a resistance/nodulation/cell division (RND)-type copper ion efflux complex (the Cus complex) (Petersen and Moller 2000; Rensing et al. 2000; Franke et al. 2003). Analysis of the M. capsulatus (Bath) genome reveals several elements that may relate to processing of copper.

The genome encodes a putative nonribosomal peptide synthetase (NRPS) that may scavenge copper; another methylotrophic bacterium *(Methylosinus)* is known to excrete copper-binding compounds (DiSpirito et al. 1998; Tellez et al. 1998). The NRPS comprises a starting module (MCA2107) containing an adenylation domain (probably recognizing a 5-hydroxy ornithine residue or another derivative of ornithine), a thiolation domain, and an unusual acetyltransferase domain. The starting module may interact with a second module (MCA1883) that contains a condensation domain and a terminal thioesterase needed for peptide release, leading to synthesis of a heavily charged peptide that could be involved in binding/scavenging of copper or other metals.

A single polyketide synthase gene (PKS) (MCA1238) is found adjacent to a gene encoding a sensor protein with diguanylate cyclase and diguanylate phosphodiesterase activities (MCA1237), often found in environmental sensing proteins and H^+^/heavy metal cation antiporters. The two-module and six-domain organization of this PKS is atypical; it contains domains with unknown functions, and its role is difficult to predict. A cation membrane transport system (MCA1900, MCA1907, MCA1911, and MCA1915) is located near the NRPS, and the 4′-phosphopantetheinyl transferase needed for activation of both PKS and NRPS is also present (MCA1522), indicating that these multimodular enzymes may be active.

M. capsulatus (Bath) has a large repertoire of 12 P-type cation ATPases, including multiple predicted copper ion pumps, which correlates with the role of copper in regulation of methane oxidation in this organism. There are also 18 resistance/nodulation/cell division–type metal ion and drug efflux pumps; the large number of these pumps, together with a variety of other metal cation uptake and efflux systems, highlight the significance of metal ion homeostasis in M. capsulatus.

The genome encodes three homologs (MCA0705, MCA0805, and MCA2072) of P-type ATPases with the characteristic copper-binding P-type ATPase motif (Solioz and Stoyanov 2003), which makes them likely to function like CopAs from other species. In Escherichia coli, CopA is regulated together with CueO, a multicopper oxidase, by CueR, a member of the MerR-family transcription regulators. No evidence for a CueR homolog was found, indicating a different mechanism of regulation of the *copA* and *cueO* genes in M. capsulatus. The genome encodes one potential *cusCBA* gene cluster (MCA2262–2264), with the *cusA* candidate (encoding the central transport protein CusA) having the same copper-binding and transport motif found in the E. coli gene. No indication of homologs of the CusF periplasmic copper chaperone was found. However, it is interesting to note that the *cusB* candidate (which encodes the CusB outer membrane protein) carries the metal-binding motif typically found in CusF, suggesting that the putative CusB may have a dual function as CusF. No evidence for the CusRS two-component response system regulating the *E. coli cusCFBA* operon was found in M. capsulatus (Bath).

In summary, there are elements of previously studied copper transport and regulation systems in the genome of M. capsulatus (Bath); the lack of the same full complement of genes and identifiable regulators raises questions about the exact operation of copper regulation and suggests future experiments to resolve this central mechanism.

#### Possible pathways for capsule biosynthesis.

As evidenced by its specific epithet, M. capsulatus possesses an insoluble polysaccharide capsule (Whittenbury et al. 1970), the composition of which has not previously been determined. Genome analysis reveals several possible pathways for the synthesis of capsular material, including colanic acid and alginate. The former includes putative colanic acid biosynthesis glycosyl transferases (MCA2124 and MCA1168), the DnaJ-like protein DjlA (MCA0020), which interacts with DnaK to stimulate colanic acid capsule synthesis (Genevaux et al. 2001), and guanine diphosphate-mannose 4,6-dehydratase (MCA1146). Also present are *rfb* genes involved in the synthesis of O antigen; O antigen can serve as capsular material, but given its additional role in lipopolysaccharide synthesis, this cannot be determined with certainty. Colanic acid is generally not produced at temperatures higher than 30 °C in E. coli (Whitfield and Roberts 1999), so alginate and O antigen may constitute capsular material at the higher growth temperatures favored by *M. capsulatus. M. capsulatus* (Bath) is somewhat desiccation resistant (Whittenbury et al. 1970), and there is evidence that desert soil methanotrophs can survive long periods of water deprivation (Striegel et al. 1992); capsule biosynthesis may aid in this.

#### Primitive pathway for asparaginyl- and glutaminyl-tRNA synthesis.

In common with the genomes of Archaea and some Bacteria, the M. capsulatus (Bath) genome lacks genes for asparaginyl-tRNA synthetase and glutaminyl-tRNA synthetase. However, a heterotrimeric glutamyl-tRNA amidotransferase (MCA0097–0099) is present, suggesting that a single amidotransferase forms asparaginyl-tRNA and glutaminyl-tRNA by transamidation of mischarged aspartyl-tRNA or glutamyl-tRNA, as found previously in many Gram-positive and some Gram-negative bacteria, archaea, and eukaryal organelles (Ibba et al. 1997; Curnow et al. 1998; Becker et al. 2000; Raczniak et al. 2001; Salazar et al. 2001). This indirect transamidation pathway has been proposed as the more ancient route to Gln-tRNA^Gln^ formation (Curnow et al. 1997), because glutamine is thought to be among the last amino acids to be added to the current repertoire of 20 amino acids. It has also been suggested that when this indirect transamidation pathway is the primary source of Gln-tRNA^Gln^ within the cell, it acts as a regulatory mechanism for glutamine metabolism (Curnow et al. 1997).

### Evidence for Evolution of Genomic Novelty

#### Genomic redundancy.

The genome of M. capsulatus (Bath) exhibits redundancy in many pathways, as described in more detail in the relevant sections above. The redundant genes fall into two categories. The first category comprises those that appear to be lineage-specific duplications (see Table 2), identified as genes encoding proteins with better BLASTP matches to other proteins in M. capsulatus (Bath) than to all other complete genomes. In some cases, these genes are found adjacent to each other in the genome, implying that they may have been generated by a tandem duplication, and that they may be transient (tandem arrays are prone to deletion). Other lineage-specific duplications, including those of many genes encoding hypothetical and conserved hypothetical proteins, may not simply be transient mutations and may instead have been maintained because they confer an evolutionary advantage on the organism. The second category contains redundant genes that do not appear to be recently duplicated, and are evolutionarily divergent, suggesting ancient duplications or exogenous acquisition. The divergent phylogeny of these genes is inconsistent with ancient duplication and subsequent vertical transmission, but we cannot determine the origin, direction, and exact path of a possible lateral transfer. Past exchange of genetic material between a methanogen and a methanotroph ancestral to *M. capsulatus,* whether direct or indirect, is certainly plausible, given their biochemical dependency. The presence of both categories of redundancy for a given enzyme or pathway makes it more plausible that the enzyme or pathway is functionally important and that its redundancy is advantageous to the organism.

*M. capsulatus,* like some other bacteria (Karunakaran et al. 2003), contains multiple copies of the chaperonins GroES and GroEL (see Table 2) that appear to be recent duplications. The two GroEL genes found in an operon structure with GroES share more sequence similarity with each other than either does to the third distal GroEL. MCA1202 is part of the *mmo* operon and was previously identified as a GroEL (Csaki et al. 2003).

The genome also contains two sets of ATP synthase genes, as has been found in four other completed genomes (*Chlorobium tepidum, Pirellula* [1], and two *Listeria* spp.), one of which is located at the putative origin of replication. Only one of these genes, that encoding the ATP synthase F_1_ epsilon subunit, appears to be recently duplicated (see Table 2), and has not been reported to be present in more than one copy in other genomes. This subunit is thought to regulate the H^+^/ATP ratio (Jones et al. 1998); it is possible that M. capsulatus alters the H^+^/ATP ratio by two different ATP synthases depending on its growth substrate (methane or glycogen/sugars). Phylogenetic analysis suggests that the other ATP synthase genes are not recently duplicated. Genes of the operon located at the origin of replication (MCA0006–0013) are of a type found only in Gammaproteobacteria or Betaproteobacteria, whereas the nonorigin genes (MCA2699–2708 and MCA1556) are divergent and related to the methanogenic Archaea and C. tepidum. The cooccurrence of the divergent genes in another organism able to fix nitrogen *(C. tepidum)* suggests that they may be involved in generation of extra ATP required to fix nitrogen.

Other redundant genes with divergent phylogenies include those that encode the MetK S-adenosylmethionine synthetase (MCA0450 and MCA0139), which is involved in methionine and selenoamino acid metabolism and has a role in activation of formate dehydrogenase; the GlpG glycogen phosphorylase (MCA0067 and MCA2540), which has a role in starch and sucrose metabolism; and the cell division protein FtsH (MCA0851 and MCA1848), which is a proteolytic regulator of cell division under stress. One of each duplicated pair is most closely related to genes from other Gammaproteobacteria, whereas its partner is either most closely related to genes from cyanobacteria, or occupies a deep-branching position.

The genome encodes formylmethanofuran dehydrogenase (MCA2860), an enzyme central to methanogenesis in Archaea. The fact that methanogenesis has not been previously reported in M. capsulatus suggests that this enzyme (along with methenyl-THMPT cyclohydrolase and formylmethanofuran THMPT formyltransferase) is instead functioning in reverse, in THMPT-linked formaldehyde oxidation (Pomper and Vorholt 2001) (Figure 2), as seen in some methylotrophs. Genes encoding subunits A, B, and C are found in an operon structure (MCA2857–2860), and there is a second distal subunit A gene (MCA2319) upstream of *pmoCAB,* together with *ftr,* which encodes the previous step in the TMPT pathway (Figure 2), which appears to be a recent duplication (see Table 2). Subunits A and C were previously known in M. capsulatus (Vorholt et al. 1999). Other genes similar to those of Archaea include those containing archaeal inteins (described above), His A/His F (involved in histidine biosynthesis in Archaea) (MCA2867), a putative arsenite transporter (MCA0791), and four conserved hypothetical proteins (MCA0196, MCA0197, MCA2834, and MCA2732).

#### Non-homology-based functional prediction.

Phylogenetic profiling (Pellegrini et al. 1999; Eisen et al. 2002) and comparative analysis of M. capsulatus (Bath) with the incomplete genome data from the methylotroph M. extorquens were used to identify additional novel genes. Phylogenetic profiling identified four genes not previously known to have a role in methane oxidation pathways in *M. capsulatus.* Two of them (MCA0180 and MCA3022) clustered with the gene that ecodes methylene THF dehydrogenase (transfer of C1 compounds) together with a gene from *Pirellula,* and the others (MCA0346 and MCA2963) grouped with *pmoC3* (oxidation of methane to methanol) and a gene from *Nitrosomonas*.

Specific comparisons with *M. extorquens,* which possesses a much larger genome (7.6 Mb) than that of M. capsulatus (Bath) (Chistoserdova et al. 2003), revealed shared genetic elements for methylotrophy. Determination of putative orthologous genes shared between M. capsulatus (Bath) and M. extorquens (best hits) yielded a total of 572 genes in 88 role categories. The majority of these shared genes are of unknown function. Putative orthologs detected included 24 conserved hypothetical genes. Phylogenetic profiling showed that ten of the 24 occur in a species distribution similar to proteins of the methane oxidation pathway, suggesting that they may also have a role in methane oxidation. Three of the ten (MCA1278, MCA1279, and MCA2862) were found within methanotrophy gene “islands” (see below), and three had the highest levels of similarity to M. extorquens (MCA1497, MCA1647, and MCA2862) supporting a putative role in the methane oxidation pathway.

Of the 89 genes putatively involved in methylotrophy in *M. extorquens,* we found orthologs of 69, mostly in the categories of energy and carbon metabolism; the remaining 20 genes found in M. extorquens but not M. capsulatus (Bath) are involved in the metabolism of other C1 compounds not used by *M. capsulatus.* Many (41 of 69) of these shared methylotrophy genes are clustered on the chromosome into 13 groups, seven of which contain more than three genes, and the largest of which contains nine. Three hypothetical proteins were identified in these clusters, suggesting a role in C1 metabolism.

### Conclusions

Our analysis of the M. capsulatus (Bath) genome has illuminated the genomic basis for the highly specialized methanotrophic lifestyle, including redundant pathways involved in methanotrophy and duplicated genes for essential enzymes such as the MMOs. We used phylogenomic analysis, gene order information, and comparative analysis with a partially sequenced methylotroph to detect genes of unknown function likely to be involved in methanotrophy and methylotrophy. Many methylotrophy genes were found to be clustered in gene islands in both organisms. We found genomic evidence for the organism's ability to acquire copper (including a previously unknown NRPS) and to use copper in regulation of methanotrophy, but the exact regulatory mechanisms remain unclear.

The genome sequence suggests previously unexpected metabolic flexibility, including the ability to oxidize chemolithotrophic hydrogen and sulfur and to live under reduced oxygen tension, both of which have implications for methanotroph ecology. There is a clear need for experimental validation of these genome-based hypotheses.

The availability of the complete genome of M. capsulatus (Bath) deepens our understanding of methanotroph biology, its relationship to global carbon cycles, and its potential for biotechnological applications, and it provides a set of hypotheses of gene function that can now be experimentally tested. In addition, the annotated genome provides a source of gene probes for detection and differentiation of methanotrophs in environmental samples.

## Materials and Methods

### 

#### Genome sequencing

M. capsulatus (Bath) was purchased from National Collection of Industrial and Marine Bacteria (Aberdeen, United Kingdom) as strain NCIMB 11132, and its DNA was isolated as previously described (Johnson 1994). The complete genome sequence was determined using the whole-genome shotgun method (Venter et al. 1996). Clone libraries with insert sizes of 1.8–2.8 kb (small) and 6.5–11 kb (medium) were used for the random shotgun-sequencing phase. Physical and sequencing gaps were closed using a combination of primer walking, generation and sequencing of transposon-tagged libraries of large-insert clones, and multiplex PCR (Tettelin et al. 1999). Sequence assembly was performed using The Institute for Genome Research (TIGR) Assembler (Sutton et al. 1995). Repeats were identified using RepeatFinder (Volfovsky et al. 2001), and sequence and assembly of the repeats were confirmed using medium-insert clones that spanned the repeat.

#### Sequence annotation

Identification of putative protein-encoding genes and annotation of the genome were performed as previously described (Eisen et al. 2002). An initial set of open reading frames predicted to encode proteins (also termed CDSs here) was initially identified using GLIMMER (Salzberg et al. 1998a). Open reading frames consisting of fewer than 30 codons and those containing overlaps were eliminated. Frame shifts and point mutations were corrected or designated “authentic.” Functional assignment, identification of membrane-spanning domains, determination of paralogous gene families, and identification of regions of unusual nucleotide composition were performed as previously described (Tettelin et al. 2001). Phylogenomic analysis (Eisen 1998a, 1998b; Eisen and Fraser 2003) was used to assist with functional predictions. Initially, all putative M. capsulatus (Bath) proteins were analyzed using the Automated Phylogenetic Inference System (J. H. Badger, personal communication, 2003). This system automates the process of sequence similarity, alignment, and phylogenetic inference for each protein in a genome. Sequence alignments and phylogenetic trees were refined using the methods described previously (Salzberg et al. 2001; Wu et al. 2004).

#### Comparative genomics

Proteins were searched by BLASTP (Altschul et al. 1990) against the predicted proteomes of published complete organismal genomes and a set of complete plastid, mitochondrial, plasmid, and viral genomes. The results of these searches were used (a) for phylogenetic profile analysis (Pellegrini et al. 1999; Eisen and Wu 2002), (b) to identify putative lineage-specific duplications (proteins showing the highest E-value scores in pairwise comparison to another protein from M. capsulatus [Bath]), and (c) to determine the presence of homologs in different species. Orthologs between the M. capsulatus (Bath) genome and that of M. extorquens were identified by requiring mutual best-hit relationships (E-values less than 10^–15^) among all possible pairwise BLASTP comparisons, with some manual corrections. A total of 89 genes involved in methylotrophy in M. extorquens (Chistoserdova et al. 2003) were obtained from GenBank and used in a BLASTP search against M. capsulatus (Bath) and M. extorquens. Comparative genome analyses were also performed using the Comprehensive Microbial Resource (Peterson et al. 2001).

#### Identification of prophage regions

Putative prophage regions were defined as containing genes that encode proteins bearing sequence similarity to known phage or prophage proteins. We are using “prophage” to refer to sequences with similarity to lysogenic bacteriophages that have not been experimentally demonstrated to form infectious particles. Additional supporting information included the presence of direct repeats representing the att_core_ of the putative prophage (identified using MUMmer [Kurtz et al. 2004]), the conserved late-gene operon responsible for packaging and head morphogenesis of tailed dsDNA bacteriophages (Duda et al. 1995), and, in the case of bacteriophage Mu-like phages, conserved gene order (putative phage repressor, transposase A and B subunits, and a Mu-like *mom* DNA methyltransferase) demarking the 5′ and 3′ boundaries of the region (Morgan et al. 2002). Best matches were determined by searching a custom database containing 14,585 total amino acid sequences from 185 published completed bacteriophage genomes, one TIGR unpublished completed bacteriophage genome, five published incomplete bacteriophage genomes, 54 published prophage genomes, and 18 TIGR unpublished putative prophage genomes, for a total of 258 unique phage or prophage entries. WU-BLASTP version 2.0 (Altschul et al. 1990) was implemented through an in-house modification of the Condor parallel search tool (Litzkow et al. 1988), reporting only those hits having E-values less than or equal to 10^–6^. In-house Perl and Linux shell scripts were used to identify the best hit (lowest E-value) per protein sequence query.

## Supporting Information

### Accession Number

The GenBank (http://www.ncbi.nlm.nih.gov/Genbank/) accession number for the Methylococcus capsulatus genome discussed in this paper is AE017282.

## Abbreviations

ABC: ATP-binding cassette
ACP: acyl carrier protein
CDS: coding sequence
G+C percent: DNA base composition (mol% guanine + cytosine)
MMO: methane monooxygenase
NRPS: nonribosomal peptide synthase
PKS: polyketide synthase
pMMO: particulate form of MMO
RuMP: ribulose monophosphate
sMMO: soluble form of MMO
SQR: sulfide-quinone reductase
TCA: tricarboxylic acid
THF: tetrahydrofolate
THMPT: tetrahydromethanopterin
TIGR: The Institute for Genome Research
